# Inferring a Causal Relationship between Environmental Factors and Respiratory Infections Using Convergent Cross-Mapping

**DOI:** 10.3390/e25050807

**Published:** 2023-05-17

**Authors:** Daipeng Chen, Xiaodan Sun, Robert A. Cheke

**Affiliations:** 1 School of Mathematics and Statistics, Xi’an Jiaotong University, Xi’an 710049, China; chendaipeng@stu.xjtu.edu.cn; 2 Mathematical Institute, Leiden University, 2333 CA Leiden, The Netherlands; 3 Natural Resources Institute, University of Greenwich at Medway, Central Avenue, Chatham Maritime, Chatham ME4 4TB, Kent, UK; robert.cheke@btinternet.com

**Keywords:** environmental factors, respiratory infection, nonlinear system, causality

## Abstract

The incidence of respiratory infections in the population is related to many factors, among which environmental factors such as air quality, temperature, and humidity have attracted much attention. In particular, air pollution has caused widespread discomfort and concern in developing countries. Although the correlation between respiratory infections and air pollution is well known, establishing causality between them remains elusive. In this study, by conducting theoretical analysis, we updated the procedure of performing the extended convergent cross-mapping (CCM, a method of causal inference) to infer the causality between periodic variables. Consistently, we validated this new procedure on the synthetic data generated by a mathematical model. For real data in Shaanxi province of China in the period of 1 January 2010 to 15 November 2016, we first confirmed that the refined method is applicable by investigating the periodicity of influenza-like illness cases, an air quality index, temperature, and humidity through wavelet analysis. We next illustrated that air quality (quantified by AQI), temperature, and humidity affect the daily influenza-like illness cases, and, in particular, the respiratory infection cases increased progressively with increased AQI with a time delay of 11 days.

## 1. Introduction

Understanding the driving force of infectious disease spread is critical to designing effective interventions curbing the threat of diseases transmission to public health around the world [1]. In tracking driving factors of infectious disease spread, examining the relationship between environmental changes and disease transmission is one of the most important topics [2,3,4,5]. Previous works have established that monsoon rains and temperature affect the epidemiology of cholera [6] and the life cycles of vectors such as mosquitoes, and the parasites that they transmit, so they are important environmental drivers of malaria [7], dengue [8] and Ross River fever [9]. Moreover, regional temperature and humidity are also related to influenza transmissibility [10,11]. Recently, there is an increasing recognition that poorer air quality is synchronized with a higher incidence of infectious diseases [12]. For example, air pollution is associated with an increased risk of tuberculosis [13,14], influenza [15,16,17], influenza-like illness [18,19] and COVID-19 [20,21]. Unlike the vector-transmitted diseases [7,8,9] with clear biological evidence, however, the driving effect of environments on respiratory infection is still controversial, at least partly because two unlinked variables in complex systems may have significant correlations [22]. To design interventions which can reduce infection risk effectively, it is of great importance to infer or falsify causal links between environmental factors and respiratory infection.

Recent studies have revealed a broad correlation between respiratory infections and environmental factors during climate change [23]. While correlation does not imply causality [24], correlated variables may potentially share information in a complex system and increase the complexity of this system. To address the issue that a system is too complex to be parameterized, researchers have developed a nonparametric framework called empirical dynamic modeling (EDM) that is designed to analyze complex systems using observed time series [25,26,27,28,29]. In the EDM methods, convergent cross-mapping (CCM) is specifically used to detect causal relationships in complex systems [28]. This approach is based on the mathematical theory of reconstructing attractor manifolds [30,31], that is, in the homeomorphic sense, the attractor of a dynamical system can be reconstructed from the time series of a single observed variable of this system. Therefore, the reconstructed manifolds of two bidirectionally coupled variables are homeomorphic [30]. For two unidirectionally coupled variables, the reconstructed manifold of the response variable is homeomorphic to the original attractor, while the reconstructed manifold of the driving variable is only a subset of the original attractor and is therefore a subset of the reconstructed manifold of the response variable [31]. Based on these consequences, Sugihara et al. [28] developed convergent cross-mapping (CCM) to predict the points in one reconstructed manifold using the points in another reconstructed manifold to then infer/falsify a causal relationship between two variables through the accuracy of predictions (CCM skill). To date, CCM has been used to analyze the causality involved in a prey–predator system [28], Earth system [32], locust abundance [33], and deep-sea biodiversity [34].

**Figure 1:**
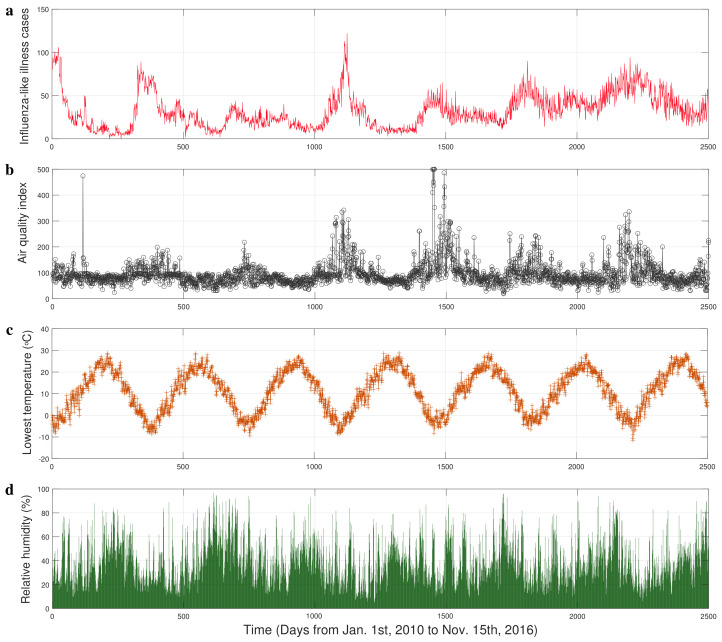
The time series of influenza-like illness (ILI) cases and experimental factors in Xi’an. (**a**) The influenza-like illness (ILI) cases collected from seven hospitals. (**b**) The real time air quality index (AQI). (**c**) The lowest daily temperature. (**d**) The relative humidity.

Although CCM has been widely applied to infer causality between variables with weak to moderate coupling strengths [28,32,33,34], Sugihara et al. [28,35] found that it is very easy to infer two variables with an unidirectionally strong coupling as having two-way causality because of the phenomenon of “generalized synchrony” [36]. To resolve this problem, Ye et al. [35] extended the CCM by considering the time delay between interacting variables. In contrast to the CCM where the predicted values of one of two variables is based on the values of another with the same time label, the extended CCM takes into account that the value of the driving variable should be more suitable for predicting the future values of the response variable and that the response variable is better at predicting the past values of the driving variable [35]. The time delay with optimal prediction suggests a causal relationship between two variables and gives an estimate of the interaction lag [35]. The biggest challenge for extended CCM is that the optimal time delay is not unique when the observation data present periodicity [37]. For respiratory infections such as influenza [38] and influenza-like illness (Figure 1a), an annual cycle is one of the most obvious characteristics. Thus, extended CCM appears to be limited by its potential inability to infer causality between respiratory infections and environmental factors.

Here, we theoretically demonstrated that while the time lags that make the CCM skill locally optimal are not unique, they occur periodically, and the period has a lower bound. This inspired the notion that the time delay with optimal prediction is unique in a narrow testing window whose width does not exceed the lower bound. The numerical analysis on a mathematical model is consistent with our theoretical analysis. For real data in Xi’an, our analysis shows that air quality, temperature, and humidity are driving factors of respiratory infection with different time delays, and suggests that the interventions such as improving air quality and appropriately increasing temperature as well as humidity could reduce the respiratory infection risk in the population.

## 2. Materials and Methods

### 2.1. The Data

The data of respiratory infections (Figure 1a) we used consist of reports of daily cases who seek medical attention with influenza-like illness (ILI) (symptoms commonly include fever, shivering, chills, malaise, dry cough, loss of appetite, body aches, and nausea) in Xi’an from 1 January 2010 to 15 November 2016 [19]. Air pollution is a mixture of multiple pollutants, so an air quality index (AQI) is used by government agencies to communicate to the public the pollution levels of the air. [39]. The AQI in Xi’an (Figure 1b) was collected from the website [40] which is an open platform for weather data. The time series of temperature and relative humidity (Figure 1c,d) were downloaded from the shared portal [41] of the China Meteorological Administration.

**Figure A1:**
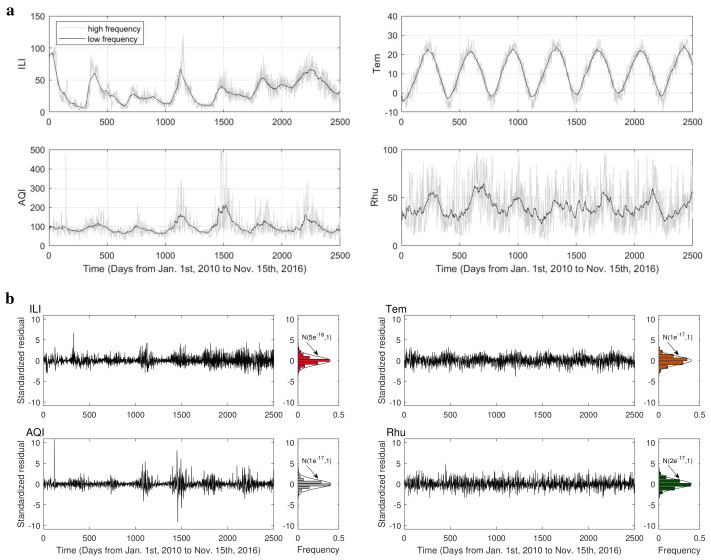
Results of Kalman filtering. (**a**) Data of the influenza−like illness (ILI) cases, air quality index (AQI), lowest daily temperature and relative humidity, low−frequency component, and original date (high−frequency component). (**b**) The time series and distribution of standardized residuals. The residuals were calculated by subtracting low−frequency component from the original series.

An alternative test for causality is the Granger test [42], but this method is inappropriate for nonlinear dynamic systems [43], so the convergent cross-mapping was proposed by Sugihara et al. [28]. One of the limitations of CCM is that CCM is sensitive to high levels of process noise in the data [44]. In order to reduce the noise level, we split the time series of collected data into low-frequency series and residuals (Figure A1a) using Kalman filtering [45]. Since the standardized residuals follow the normal distribution (Figure A1b), we assumed that the main information in real time series was included in the filtered low-frequency series which is used in the following analysis.

### 2.2. The Method

The general dynamical system actually corresponds to a complex causal network of interlocking variables. We apply extended convergent cross-mapping (CCM) [28,35] to examine the causality and interaction delay between two variables. We let x(t) and y(t) be the observed time series corresponding to the variables x and y, respectively, and begin by reconstructing the lagged-coordinate vectors X(t)=[x(t),x(t−s),⋯,x(t−(Π−1)s)] and Y(t)=[y(t),y(t−s),⋯,y(t−(Π−1)s)] with dimension Π based on the Takens embedding theorem [30,31]. We denote the reconstructed manifolds as $M_{x}=\left\{X\left(t\right)\right\}$ and $M_{y}=\left\{Y\left(t\right)\right\}$. For any point $Y(t_{*})$ in $M_{y}$, we mark the time label of its Π+1 nearest neighbors in $M_{y}$ as $t_{1},t_{2},\cdots ,t_{\Pi +1}$, and the estimated point $\bar{X}\left(t_{*}+\tau \right)$ with some delay τ on $M_{x}$ is given by the simplex projection [25]:

(1) $$\bar{X}\left(t_{*}+\tau \right)=\sum_{i=1}^{\Pi +1}\chi^{i}X\left(t_{i}+\tau \right),$$

where

$$\chi^{i}=\frac{exp(-∥(Y\left(t_{i}\right)-Y\left(t_{*}\right)∥/∥Y\left(t_{1}\right)-Y\left(t_{*}\right)∥)}{\sum_{i=1}^{\Pi +1}exp(-∥(Y\left(t_{i}\right)-Y\left(t_{*}\right)∥/∥Y\left(t_{1}\right)-Y\left(t_{*}\right)∥)}.$$

We denote $\bar{x}\left(t|\tau \right)$, the first coordinate of $\bar{X}\left(t\right)$, as the estimated value of time series x(t) using this method. We use the Pearson correlation coefficient $\rho \left(\tau \right)=Corr(\bar{x}\left(t|\tau \right),x\left(t\right))$ between estimated values and observed values to quantify CCM skill. From the perspective of the extended CCM [35], the CCM skill ρ(τ) reaching its maximum at a negative τ means that there is a driving force from x to y with time delay |τ|.

Given a *T*-periodic observed time series x(t), the reconstructed manifold $M_{x}$ is a closed orbit in the Π-dimensional space. According to the Whitney embedding theorem [46], Π=3 is sufficient to ensure that all information of the original complex system is represented in the periodic orbit $M_{x}$. During any small period [t,t+Δt], the length Δl(t) of the corresponding small arc on $M_{x}$ can be approximated by

(2) $$\Delta l\left(t\right)=\sqrt{x^{\prime }{\left(t\right)}^{2}+x^{\prime }{\left(t-s\right)}^{2}+x^{\prime }{\left(t-2s\right)}^{2}}\Delta t.$$

The prediction using simplex projection (1) mainly depends on the local distance between points [25] on the reconstructed manifold $M_{x}$. Therefore, the characters of Δl(t) affect the evaluation accuracy of extended CCM. If *s* is very small (i.e., the data are collected densely), then $\Delta l\left(t\right)\approx \sqrt{3}∥x^{\prime }\left(t\right)∥\Delta t$. To investigate the mathematical characters of Δl(t) (i.e., $∥x^{\prime }\left(t\right)∥$), as the first step, we give the following proposition.

 **Proposition 1.**  *A continuous, periodic, and nonconstant function x(t) has a smallest (positive) period T. For any other period $\tilde{T}$ of x(t), there is an integer n such that $\tilde{T}=nT$.*

 **Proof.**  Suppose there is no smallest (positive) period, then there is a decreasing (positive) sequence $T_{1}$, $T_{2}$,…,$T_{n}$,… of periods such that $\lim_{n\to \infty }T_{n}=0$. For any given $t_{0}$ and $t_{1}$ ($t_{0}<t_{1}$), we define a sequence of integers:

$$z_{1}=\left[\frac{t_{1}-t_{0}}{T_{1}}\right],z_{2}=\left[\frac{t_{1}-t_{0}}{T_{2}}\right],\cdots ,z_{n}=\left[\frac{t_{1}-t_{0}}{T_{n}}\right],\cdots$$

then the sequence $t_{1}$, $t_{n+1}=t_{n}-z_{n}T_{n}$ (n=1,2,⋯) such that

$$x\left(t_{n}\right)=x\left(t_{n+1}\right)\;\mathrm{and}\;\lim_{n\to \infty }t_{n}=t_{0}.$$

By continuity of x(t), $x\left(t_{1}\right)=\lim_{n\to \infty }x\left(t_{n}\right)=x\left(t_{0}\right)$, which contradicts that x(t) is a nonconstant function. Consequently, there is a smallest (positive) period *T* of x(t).

For any other period $\tilde{T}$ of x(t), there always is an integer *k* such that $kT<\tilde{T}\leq \left(k+1\right)T$. Consequently,

$$0<\tilde{T}-kT\leq T.$$

If $\tilde{T}\neq \left(k+1\right)T$, then $\tilde{T}-kT$ is a new smallest (positive) period of x(t) because

$$x\left(t+\tilde{T}-kT\right)=x\left(t\right).$$

Therefore, there is an integer n=k+1 such that $\tilde{T}=nT$ for any other period $\tilde{T}$ of x(t). □

Based on the result of Proposition 1, we further draw the following proposition about the derivative of a nonconstant periodic function.

 **Proposition 2.**  *For any smooth periodic function x(t), the derivative $x^{\prime }\left(t\right)$ is a periodic function and minimum period of $x^{\prime }\left(t\right)$ is the minimum period T of the original function.*

 **Proof.**  On the one hand,

$$x^{\prime }\left(t\right)=\lim_{▵t\to 0}\frac{x(t+▵t)-x(t)}{▵t}=\lim_{▵t\to 0}\frac{x(t+T+▵t)-x(t+T)}{▵t}=x^{\prime }\left(t+T\right).$$

Therefore, $x^{\prime }\left(t\right)$ is a periodic function. Suppose the minimum period of derivative $x^{\prime }\left(t\right)$ is $\bar{T}$, and then Proposition 1 implies that there is a positive integer $k_{1}$ such that $T=k_{1}\bar{T}$. On the other hand,

$$x^{\prime }\left(t\right)=x^{\prime }\left(t+\bar{T}\right)$$

gives

$$\int_{t}x^{\prime }\left(s\right)ds=\int_{t}x^{\prime }\left(s+\bar{T}\right)ds=\int_{t+\bar{T}}x^{\prime }\left(s\right)ds.$$

Consequently, $x\left(t+\bar{T}\right)=x\left(t\right)+C$ for some constant *C*. Because function x(t) is periodic, we must have C=0. Therefore, there is a positive integer $k_{2}$ such that $\bar{T}=k_{2}T$. Collectively, $k_{1}=k_{2}=1$. □

Finally, we give a proposition which presents the mathematical characters of the length Δl(t) (i.e., $∥x^{\prime }\left(t\right)∥$) of the corresponding small arc on the reconstructed manifold $M_{x}$ from time series x(t).

 **Proposition 3.**  *Given a smooth function x(t) with minimum period T, suppose that the function has at most k extreme points in a single period, then the function $∥x^{\prime }\left(t\right)∥$ is periodic and the minimum period of $∥x^{\prime }\left(t\right)∥$ is not less than T/k.*

 **Proof.**  The result of Proposition 2 yields that

$$∥x^{\prime }\left(t+T\right)∥=∥x^{\prime }\left(t\right)∥.$$

This shows that the function $∥x^{\prime }\left(t\right)∥$ is periodic. Suppose the minimum period of function $∥x^{\prime }\left(t\right)∥$ is $\ddot{T}$, and then by Proposition 1 that there is a positive integer *n* such that $T=n\ddot{T}$. For any period [t,t+T], we have

$$\int_{t}^{t+T}x^{\prime }\left(s\right)ds=x\left(t+T\right)-x\left(t\right)=0.$$

Therefore, there is a $t_{0}\in \left[t,t+T\right]$ such that $x^{\prime }\left(t_{0}\right)=0$, and then $∥x^{\prime }\left(t_{0}\right)∥=0$. Without loss of generality, we assume that $t_{0}-t<\ddot{T}$. Consequently, we have $t_{0}$, $t_{0}+\ddot{T}$, $t_{0}+2\ddot{T}$, …, $t_{0}+\left(n-1\right)\ddot{T}\in \left[t,t+T\right]$ such that

$$∥x^{\prime }\left(t_{0}\right)∥=∥x^{\prime }\left(t_{0}+\ddot{T}\right)∥=∥x^{\prime }\left(t_{0}+2\ddot{T}\right)∥=\cdots =∥x^{\prime }\left(t_{0}+\left(n-1\right)\ddot{T}\right)∥=0.$$

Thus, n≤k, which means that $\ddot{T}=T/n\geq T/k$. □

Simplex projection (1) means that the higher-density data points (i.e., smaller arc length Δl(t)) on manifold $M_{x}$ corresponds to lower uncertainty of estimates [25], which is consistent with the tests on simulated data and real data [37]. The proposition 3 links the period of arc length Δl(t) (2) to the period of time series x(t) via the function $∥x^{\prime }\left(t\right)∥$. For the time series x(t) of common environmental infectious disease with period *T*, based on Proposition 3, the minimum positive period of $∥x^{\prime }\left(t\right)∥$ is the heuristic T/2. In addition, an infectious disease such as hand–foot and mouth disease (HFMD) with multiple peaks [47], wavelet analysis [48] of corresponding time series estimates the periodicity of peaks. Collectively, for environmental infectious disease with estimated minimal period *T*, if the Pearson’s correlation coefficient ρ(τ) reaches a local maximal at $\tau_{*}$, ρ(τ) will not peak again in ($\tau_{*}-T/2$, $\tau_{*}+T/2$). Thus, we provide a boundary B=(−T/4,T/4) with width T/2 as the empirical testing window so that the extended CCM can be used to infer causality between periodic variables such as seasonal infectious diseases and environmental factors.

We next present the procedure to infer causality between variables x and y. For observed time series x(t) and y(t) with significant period *T*, the response time delay of variable *x* to variable *y* can be estimated by the following formula:

(3) $${\bar{\tau }}_{xy}={\arg\max}_{\tau \in B}Corr\left(\bar{y}\left(t|\tau \right),y\left(t\right)\right),$$

where $\bar{y}\left(t|\tau \right)$ are the estimated values of time series y(t). Similarly, we can also obtain the estimated response time delay of variable *y* to variable *x* using the formula

(4) $${\bar{\tau }}_{yx}={\arg\max}_{\tau \in B}Corr\left(\bar{x}\left(t|\tau \right),x\left(t\right)\right).$$

We consider the sign of ${\bar{\tau }}_{xy}$ and ${\bar{\tau }}_{yx}$ comprehensively to infer the causality between the variables x and y. If ${\bar{\tau }}_{xy}\geq 0$ and ${\bar{\tau }}_{yx}<0$, then variable x affects future values of y unidirectionally with time delay $-{\bar{\tau }}_{yx}$. If ${\bar{\tau }}_{xy}<0$ and ${\bar{\tau }}_{yx}\geq 0$, then variable y affects future values of x unidirectionally with time delay $-{\bar{\tau }}_{xy}$. If ${\bar{\tau }}_{xy}<0$ and ${\bar{\tau }}_{yx}<0$, then x and y have two-way cause and effect, and the action time delay is $-{\bar{\tau }}_{yx}$ and $-{\bar{\tau }}_{xy}$, respectively. If ${\bar{\tau }}_{xy}\geq 0$ and ${\bar{\tau }}_{yx}\geq 0$, then we conclude that there is no causal evidence between variables x and y. We choose the negative optimal cross-lag as the estimated time delay because CCM is a historical information-dominated method [28], which is also consistent with previous extension [35].

Collectively, we have made an adjustment on the basis of CCM, and this update makes up for the limitation of the extended CCM in inferring causality between periodic time series.

## 3. Results

### 3.1. Testing on an Infectious Disease Model

**Figure 2:**
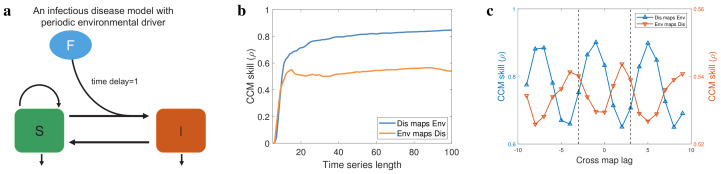
Numerical validation of theoretical results. (**a**) An environmental factor (F)−embedded susceptible−infectious−susceptible (SIS) epidemic model, in which the dynamics of environmental factor is periodic. The effect of environmental factor on disease incidence has a time delay 1. (**b**) The performance of CCM and the CCM skill as a function of the length of time series used to reconstruct the high−dimensional manifold. (**c**) The performance of extended CCM and the CCM skill as a function of the tested time delay. Here, the length of time series used to reconstruct the high−dimensional manifold is fixed.

As the first step, we test the causal inference methods on an air quality index (AQI)–embedded susceptible–infectious–susceptible (SIS) epidemic model (Figure 2a). In this model, the total population (*N*) consists of classes of individuals that are susceptible (*S*) and infectious (*I*), yielding

(5) $$\begin{array}{c} S\left(t+1\right)=f\left(N\left(t\right)\right)+\sigma \phi \left(\frac{I(t)}{N(t)},\alpha F\left(t\right)\right)S\left(t\right)+\gamma \sigma I\left(t\right),\\ I\left(t+1\right)=\sigma (1-\phi \left(\frac{I(t)}{N(t)},\alpha F\left(t\right)\right))S\left(t\right)+\left(1-\gamma \right)\sigma I\left(t\right). \end{array}$$

where *t* is time, f(N(t)) is the density-dependent birth rate or recruitment according to the formula f(N(t))=N(t)exp(r−N(t)), σ is the probability of survival, α is a modified parameter for the effect of air pollution on incidence, and γ is the recovery rate. We assume that the proportion of susceptible individuals that do not become infected at time *t* is $\phi \left(z\left(t\right),w\left(t\right)\right)=e^{-\beta z(t)}e^{-\beta w(t)}$ given the disease prevalence z(t)=I(t)/N(t) and air pollution effect w(t)=αF(t), that is, encounters leading to infection are modeled via a Poisson process with the transmission constant β. The environmental driver F(t) (i.e., air quality index) varies according to the following formula, which is a discrete time form of a continuous time system [49].

(6) F(t+1)=λ(t)F(t)+C

where λ(t)=a−b*sin(t/ω) is the remaining proportion of mixed pollutants in the air from time step *t* to t+1; *C* is the constant rate of inflow of pollutants into the air, mainly depending on the persistent release of various air pollutants. We simulated the system with parameters setting in (Appendix A.1) and generated time series of the environmental factor and infectious individuals.

We first test CCM [28] using these simulated data, and we find that the CCM skill in both direction becomes better as the length of time series increases (Figure 2b), which implies a bidirectional causality between environmental factor and disease incidence [28] instead of the true unidirectional causality in our simulated system (Figure 2a). Thus, CCM is not suitable for inferring causality in the strongly coupled systems, because the strong coupling strength leads to a synchrony between response variable and driving variable, resulting in the dynamics of a response variable becoming dominated by those of the driving variable [35]. We next apply extended CCM [35] to identify the optimal cross-map lags between environmental factor and disease. In our numerical simulations, λ(t) is a 4π-period function (Appendix A.1), so the period of time series F(t) and I(t) are approximately 12. The results of extended CCM show that the optimal time lag is not unique (Figure 2c), and the difference between two adjacent local optimal delays is around 6, which is consistent with Proposition 3 and the period of time series F(t) and I(t).

In addition, by setting B=(−3,3) as shown by the vertical dashed lines in Figure 2c, we estimated that ${\bar{\tau }}_{Dis,Env}=-1$ and ${\bar{\tau }}_{Env,Dis}=2$ using the Formulas (3) and (4). Therefore, our inference is that the environmental factor affects disease incidence with a time delay 1, which is consistent with the modeling (Figure 2a). Collectively, we showed the limitations of CCM and extended CCM in inferring the causality between periodic time series (Figure 2b,c), which are overcome by adding an estimation interval for the extended CCM (Formulas (3) and (4)).

### 3.2. Correlation Analysis and Wavelet Analysis of Real Data

**Figure 3:**
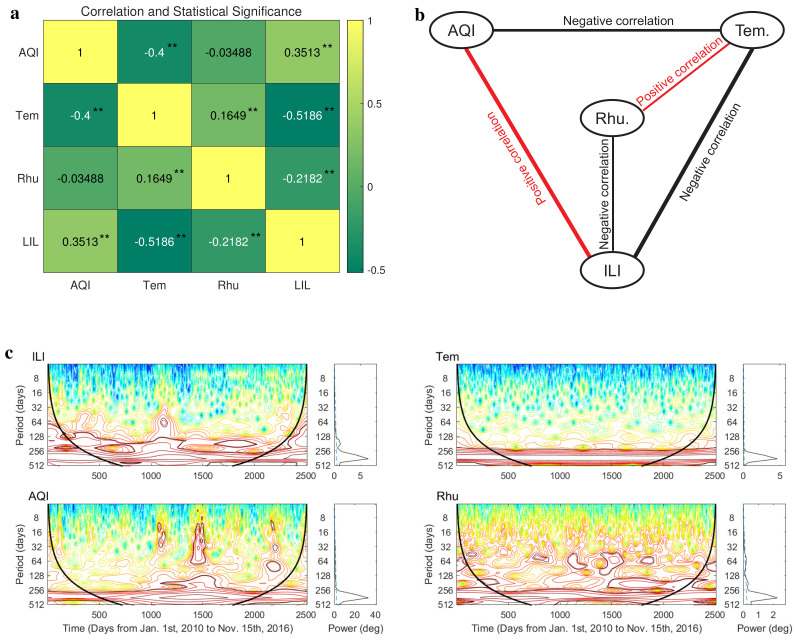
Correlation analysis and wavelet analysis of real data in Xi’an. (**a**) The correlation and statistical significance among the observed time series of influenza−like illness (ILI) cases, air quality index (AQI), lowest daily temperature, and relative humidity in Xi’an. *p*-values are < 0.01 (**). (**b**) The correlation network among the four variables we studied. (**c**) Wavelet analysis for time series of influenza−like illness (ILI) cases, air quality index (AQI), lowest daily temperature, and relative humidity. Wavelet power spectra are depicted on the left, and the right−hand panels show the mean spectra (vertical solid black line) with their significant threshold value of 0.05 (blue dashed line).

As a comparison, before inferring the causality among respiratory infection, air pollution, temperature, and humidity using the real data in Xi’an (Figure 1), we analyzed the correlation between these data and provided statistic significance of the correlation (Figure 3a). Based on the result, we constructed a correlation network which is undirected (Figure 3b). Edges in this network simply indicate that the variations in two connected variables are positively or negatively correlated, but do not distinguish between a driving variable and a response variable. In addition, we found that there is no significant correlation between air quality index (AQI) and relative humidity (Rhu) in Xi’an. According to the theoretical analysis (Proposition 3) and numerical analysis (Figure 2), the periodicity of observed time series affects the performance of extended CCM. Moreover, the minimum positive period of the time series also determines the setting of the estimation interval for the optimal time delay between two variables. Here, we investigated the periodicity of influenza-like illness (ILI) cases, air quality index (AQI), daily temperature, and relative humidity in Xi’an using the wavelet analysis ([48]; Figure 3c). The results of wavelet analysis [48] show that all time series have only a significant annual cycle (see Figure 3c), so we can detect the optimal time lag in a narrow window *B* with the width of no more than 180 days using extended CCM (Formulas (3) and (4)). In the following analysis, we detect the optimal lag in the estimation interval (−50,50).

### 3.3. Causality Analysis of Real Data

**Figure 4:**
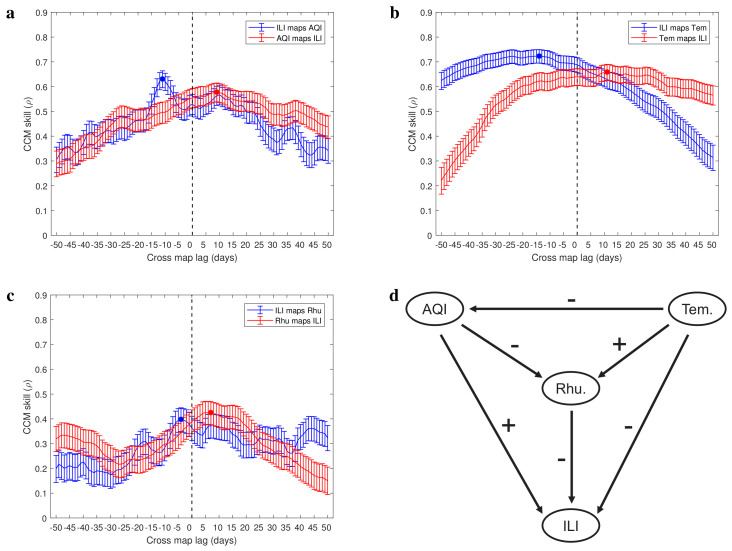
Causal evidence among four variables (ILI−influenza like illness, AQI−air quality index, Tem.−temperature and Rhu.−relative humidity). (**a**–**c**) The CCM skills between involved variables as a function of tested cross-map lag. The negative optimal cross-map lag is the estimated interaction delay between them, e.g., the estimated delay for air pollution to drive influenza−like illness cases is 11 days. (**d**) Estimated causal network. The signs associated with arrows represent positive or negative correlation between two nodes. A negative sign means that increasing the drive variable would inhibit the response variable, and a positive sign means that higher drive variable would promote the response variable.

**Figure 5:**
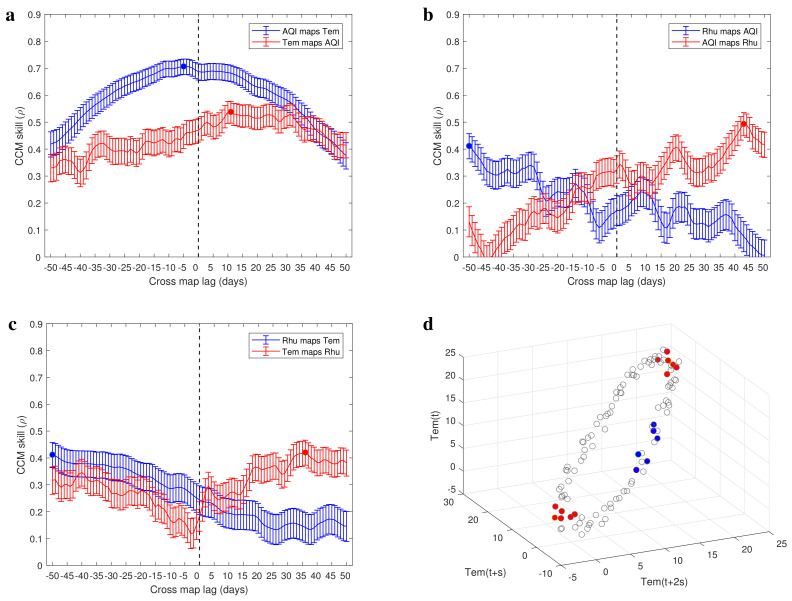
(**a**–**c**) Causal evidence between environmental factors. (**d**) Reconstructed manifold using the time series of temperature. Consistent with the theoretical analysis, the variations of points density in the reconstructed manifold is periodic (the blue points and red points are the 6 nearest neighbors of some points).

In Figure 4a–c and Figure 5a–c, we present the CCM skills using extended CCM in a narrow testing window. The cross-mapping skills between influenza-like illness and AQI time series indicate that there is a driving force from air pollution to respiratory infections with time delay of 11 days (${\bar{\tau }}_{ILI,AQI}=-11$ and ${\bar{\tau }}_{AQI,ILI}=9$; Figure 4a). The cross-mapping skills between influenza-like illness and temperature time series indicate that there is a driving force from temperature to respiratory infections with a delay of 14 days (${\bar{\tau }}_{ILI,Tem}=-14$ and ${\bar{\tau }}_{Tem,ILI}=11$; Figure 4b). Figure 4c shows the cross-mapping skills between influenza-like illness and relative humidity time series, which indicate that there is a driving force from relative humidity to respiratory infections with time delay of 4 days (${\bar{\tau }}_{ILI,Rhu}=-4$ and ${\bar{\tau }}_{Rhu,ILI}=7$). We further analyzed the causality between environmental factors (see Figure 5a–c) and obtained a causal network among the four variables studied (see Figure 4d). The sign on each side comes from the correlation analysis (Figure 3a). In contrast to the correlation network (Figure 3b), the causality network is a directed network where the edge distinguishes the driving variable and response variable (Figure 4d). From this directed network, we predict that increasing temperature increases relative humidity and decreases the air pollution degree as well as respiratory infections risk. More serious air pollution decreases relative humidity and increases respiratory infections risk, but higher relative humidity decreases respiratory infections risk. This indicates that in order to reduce the risk of respiratory infections, the indoor temperature and humidity can be improved by using air conditioners and air humidifiers.

Collectively, we inferred the causality between respiratory infections and environmental factors in Xi’an using the extended CCM by limiting the width of testing window for searching for the optimal time delay. The results enrich the studies on health effects of environmental factors.

## 4. Discussion

In this study, we presented evidence (Figure 4 and Figure 5) for a causal relationship between respiratory infection and several environmental factors such as air quality, temperature, and humidity by adopting and refining a published method, CCM (convergent cross-mapping [28]). Related to this, we first performed theoretical analysis, centered chiefly on the extended CCM [35], which supports the result that the estimated optimal time delay between driving variable and response variable is not unique as long as these variables show synchrony and periodicity [37]. In addition, our theoretical result also suggests an idea to overcome the limitation of the extended CCM in inferring causality between synchronized periodic variables, which is estimating the optimal time delay in a bounded testing window (Formulas (3) and (4)). Consistently, we illustrated the limitations of the CCM as well as the extended CCM in inferring a causal relationship, and visualized the theoretical results (Proposition 3) using the data generated from an epidemic model (Figure 2) and the real data (Figure 5d). To illustrate that the characteristics of the real data (Figure 1) are consistent with the assumptions in our theoretical result and fall within the scope of our refined approach, we evaluated the periodicity of the data using wavelet analysis (Figure 3c). The minimal period of real data determines the width of testing window for estimating the time delay between the driving variable and response variable (Formulas (3) and (4)). By performing causal analysis for the real data, we suggested that all of air quality, temperature, and humidity have an effect on the incidence of respiratory infections. In particular, taking the reporting delay into account, air pollution promotes the respiratory infections risk with a time delay of 11 days.

In China, air pollution has been a public issue for a long time [50]. In recent years, the number of respiratory infections has highly synchronized with the variations of air quality index in China [19]. Through analyzing the time series of influenza-like illness and some environmental factors in Xi’an from 1 January 2010 to 15 November 2016, one important result of our research is that air pollution fuels the risk of respiratory infections. Different from the significant correlation confirmed in previous studies [19], our study gives the causality between air pollution and respiratory infection, and identifies the detailed time delay. In addition, we found that lower temperature and humidity also fuel the respiratory infections risk. According to our estimated causal network (Figure 4d), temperature is located at the most upstream of the entire causal network and affects respiratory infections, air quality, and relative humidity. Because the temperature in the Northern Hemisphere is mainly affected by the relative position of the Earth and the sun, the variables we studied may come from a larger and more complex system.

In addition to respiratory diseases [51], air pollution also leads to 3.3 million premature deaths per year worldwide [52] and has a substantial role in many noncommunicable diseases [53] such as cancer [54], stroke [55], cardiovascular disease [56,57], and Alzheimer’s disease [58,59]. Furthermore, available evidence suggests that air pollution can prevent the beneficial cardiopulmonary effects of walking in people with heart or chronic lung disease [60,61] and results in poor lung function in children [62,63]. Therefore, our results complement previous research on the health effects of air pollution, which strengthens the importance of improving air quality.

It is well known that the variables being correlated does not imply that they are causal. Comparing the causal analysis with correlation analysis (see Figure 3b and Figure 4d), we find that the causality does not imply significant correlation either. The possible reason is that standard correlation analysis mainly captures the linear relationships between variables, while real data may arise from complex nonlinear systems in which ephemeral correlations are common [64,65]. By broadening the scope of application and improving the accuracy as we did in this study, CCM can help us understand the relationship between variables in complex systems such as molecular systems [66] and public health systems [49], in which the causality is useful for designing novel experiments and interventions, respectively.

It is worth noting that in order to ensure that the optimal cross-map lag of extended CCM is unique, we define a bounded testing window which depends on the period of time series (Formulas (3) and (4)). This means that the real time delay between driving variable and response variable should be in the testing window. Otherwise, we would give a wrong causality and a wrong time delay between two variables. Nonetheless, we are still confident that our work has a broad applicability in time series analysis. For seasonal diseases such as respiratory infections, the period of the time series is usually very long and the unit is years. Therefore, the testing window is wide enough to cover the real time delay between driving variable and response variable. In addition, due to the temporal decay of information in transfer process, when the extended CCM is used to infer causality, only a rough approximation of the action time delay can be estimated. In some cases, the estimates of action time delay may not be reliable (Figure 5b,c). Related to this, we inferred causality by following the rule that the values of the driving variable are better to estimate the future value of the response variable, whereas the values of the response variable are better to estimate the past value of the driving variable [35].

In summary, we refined a published method (CCM) for causal inference through theoretical analysis. Using this refined method to analyze the time series of influenza-like illness, air quality, temperature, and humidity in Xi’an, we established a causal network where the nodes are these variables. In this network, air pollution promotes respiratory infections risk while higher temperature and humidity limit the risk (Figure 4d). It will be of interest to test the robustness of this causal network using different datasets, and to determine how these results will impact the design of novel interventions against respiratory infections in populations.

## Data Availability

Data sources are already included in the main text.

## Abbreviations

The following abbreviations are used in this manuscript:

Tem	Tempreture
AQI	Air quality index
Rhu	Relative humidity
ILI	Influenza–like illness

## Appendix A

### Appendix A.1. The Generation and Analysis of Simulated Data

The system (5) is initialized as S(1)=5, I(1)=4, and F(1)=5, and run for 3500 time steps with the parameters λ(t)=0.6−0.3sin(t/2),r=6,σ=0.5,α=8,β=0.01, and γ=0.01 to generate the time series of I(t) and F(t). These time series are analyzed using s=1 and Π=3 which ensure the requirements of Takens’ embedding theorem [30,31]. We first test CCM [28] using these simulated data, selecting 100 pairs of 100 vectors of random libraries over time points 101–1000, computing the cross-map skill for each pair of libraries using time series length 7–100, and averaging out the cross-map skill of 100 random libraries, respectively. We then apply extended CCM [35] to identify the optimal cross-map lags, selecting 100 pairs of random libraries of 2000 vectors over time points 101–3000, computing cross-map skill for each pair of libraries with maximum length using different cross-map lag and averaging out the cross-map skill of these random libraries, respectively.

### Appendix A.2. Kalman Filtering

Kalman filtering was achieved by using the function kalman in MATLAB (available from: https://www.mathworks.com/help/control/ref/ss.kalman.html accessed on 19 January 2021). The low–frequency component was derived first (Figure A1a) and this was subtracted from the original series (high-frequency component) to provide the residual. After standardization, we found that the residuals were fitted with a standard normal distribution (Figure A1b).
